# MyD88 and TLR9 Dependent Immune Responses Mediate Resistance to *Leishmania guyanensis* Infections, Irrespective of *Leishmania* RNA Virus Burden

**DOI:** 10.1371/journal.pone.0096766

**Published:** 2014-05-06

**Authors:** Annette Ives, Slavica Masina, Patrik Castiglioni, Florence Prével, Mélanie Revaz-Breton, Mary-Anne Hartley, Pascal Launois, Nicolas Fasel, Catherine Ronet

**Affiliations:** 1 Department of Biochemistry, University of Lausanne, Epalinges, Switzerland; 2 Department of Biochemistry, World Health Organization Immunology Research and Training center (WHO-IRTC), Epalinges, Switzerland; Centre d’Immunologie de Marseille-Luminy, CNRS-Inserm, France

## Abstract

Infections with *Leishmania* parasites of the *Leishmania Viannia* subgenus give rise to both localized cutaneous (CL), and metastatic leishmaniasis. Metastasizing disease forms including disseminated (DCL) and mutocutaneous (MCL) leishmaniasis result from parasitic dissemination and lesion formation at sites distal to infection and have increased inflammatory responses. The presence of *Leishmania* RNA virus (LRV) in *L. guyanensis* parasites contributes to the exacerbation of disease and impacts inflammatory responses via activation of TLR3 by the viral dsRNA. In this study we investigated other innate immune response adaptor protein modulators and demonstrated that both MyD88 and TLR9 played a crucial role in the development of Th1-dependent healing responses against *L. guyanensis* parasites regardless of their LRV status. The absence of MyD88- or TLR9-dependent signaling pathways resulted in increased Th2 associated cytokines (IL-4 and IL-13), which was correlated with low transcript levels of IL-12p40. The reliance of IL-12 was further confirmed in IL12AB^−/−^ mice, which were completely susceptible to infection. Protection to *L. guyanensis* infection driven by MyD88- and TLR9-dependent immune responses arises independently to those induced due to high LRV burden within the parasites.

## Introduction

In Latin America, infections with protozoan, obligate intracellular parasites of the *Leishmania Viannia* subgenus, such as *L. braziliensis*, *L. panamensis* and *L. guyanensis* give rise to cutaneous (CL), and metastasizing leishmaniasis, which includes disseminated (DCL) and mucocutaneous (MCL) leishmaniasis. Infections occur following transmission to humans via the bite of infected sand flies [1]. MCL is characterized by parasitic dissemination that promotes destructive secondary skin lesions. This metastasis may have tissue-specificity, spreading to the mucosa of mouth and nose in 5 to 10% of individuals with resolved CL [1], [2]. MCL pathology is associated with chronic hyper-inflammation, concomitant with a modulation of T helper cytokines and cytotoxic T cell activity [3]–[7]. Studies have shown that host genetic polymorphisms (such as a single base pair substitution in the IL-6 and TNF-α promoters) [8]–[10], immune-status, and parasite derived virulence factors [11] are associated with the development of clinical MCL.

Previously, we reported that elevated pro-inflammatory immune response generation in *L. guyanensis* infected macrophages relies on the recognition of high levels of an endosymbiotic dsRNA virus of the *Totiviridae* family within metastasizing *L. guyanensis* promastigotes (derived from either the hamster model or human MCL isolates). The viral dsRNA genome of the *Leishmania RNA virus* (LRV) is recognized by the TRIF-dependent TLR3 signaling pathway, which resulted in an increased secretion of inflammatory cytokines and chemokines and exacerbated susceptibility to infection [3]. Although the absence of TLR7 in macrophages decreased LRV-induced pro-inflammatory mediators *in vitro*, it showed no role in progression of leishmanial disease in our mouse model [3].

In CL, a T-helper 1 (Th1) response is associated with protection, and given by high IFN-γ production and efficient intracellular parasite killing by nitric oxide. Conversely, a Th2 response is associated with susceptibility to infection which is driven by high IL-4, and IL-13 and reduced intracellular parasite killing due to increased Arginase-1 activity [12]. Furthermore, immunosuppressive IL-10 also impairs *Leishmania* parasite disease resolution and promotes lesion persistence and high parasite burden [12]. Previous work has clearly demonstrated that, for both *L. major* and *L. (Viannia) braziliensis,* the absence of MyD88-dependent signaling pathways modulated the Th1/Th2 balance following *in vivo* footpad infection and resulted in increased susceptibility to infection [13]–[18]. Further studies described that the impaired ability of MyD88-deficient (MyD88^−/−^) mice to develop Th1-dependent immune responses was due to reduced IL-12 production [17]. This consequently elevated production of the Th2 cytokine, IL-4 and IL-13, may explain why these mice displayed reduced dendritic cell maturation and naïve T cells priming following infection [14], [15], [19]. Furthermore, a role for TLR9 was also implicated in these responses as both models displayed a transient increase in susceptibility, evidenced by an increased peak in footpad swelling, associated with elevated parasite levels [18], [20], [21].

The TLR9 signaling pathway is triggered after the recognition of foreign unmethylated CpG DNA. Its position on the internal aspect of the macrophage phagosomal compartment and plasmocytoid dendritic cells makes it especially sensitive to intracellular pathogens, such as is *Leishmania*. All Toll-like receptors rely on the MyD88 adaptor protein for signal transduction (except TLR3, which is uniquely TRIF-dependent) and may thus explain the common susceptible phenotype of MyD88^−/−^ and TLR9^−/−^ mice for *L. major* and *L. braziliensis* infection.

Given the potential application for TLR-ligands such as CpG motifs to act as adjuvants in vaccination strategies [22], [23] this study assesses if resistance to *L. guyanensis* parasite infection is mediated by MyD88, and TLR9 [22], [23]. More importantly our study evaluates if host recognition of LRV1 could impair these protective immune responses, which will provide much needed information into the applicability for TLR-ligand based adjuvants being used in vaccination strategies against leishmaniasis in Latin America.

## Materials and Methods

### Ethical Statement

All the mice experiments were approved by the Ethics and Veterinary office regulations of the state of Vaud (SAV), Switzerland. Our laboratory has the administrative authorization numbers 2113-1 and 2113-2.

### Animals

8 to 10 weeks old C57BL/6 mice were purchased from Harlan Laboratories (Netherlands). MyD88^−/−^ [24] and TLR9^−/−^ mice [25] were obtained Prof. S. Akira (Osaka University, Japan) via P. Launois (WHO-IRTC, Lausanne, Switzerland). IL12AB^−/−^ mice [26] were obtained from G. Alber (University of Leipzig, Germany). Mice were maintained under pathogen-free conditions at the animal facility of the Center of Immunity and Immunology, Lausanne (Switzerland). All mutant and deficient mice were crossed onto a C57BL/6 background for at least eight generations. Each experiment contained at least 4 mice per group.

### Parasite and Cell Culture

The *L.g.* LRV^high^ (M5313) and *L.g.* LRV^low^ (Lg17) *L. guyanensis* parasites used in this study were derived from the strain *L. guyanensis* (*L.g.* M5313(M+),WHI/BR/78/M5313) from CIDEIM (Centro Internacional de Entrenamiento e Investigaciones Médicas) [27] and LRV burden was previously determined [3]. Parasites were cultured at 23°C in M199 medium (Gibco) consisting of 10% FBS, 1% penicillin/streptomycin, and 5% Hepes (Sigma-Aldrich), or grown in freshly prepared Schneider’s Insect Medium (Sigma-Aldrich) supplemented with 10% heat-inactivated fetal bovine serum, 2 mM L-glutamine, 1% penicillin/streptomycin (Gibco).

### Mouse Infection and Parasite Quantification

3×10^6^ parasites of *L.g*. LRV^low^ (Lg17), and *L.g.* LRV^high^ (M5313) were infected into the hind footpads of mice. Weekly footpad swelling measurements were taken using a Vernier caliper. Parasite burden was quantified within the *L. guyanensis* infected footpads using the standard curve real time PCR quantification method based on *Leishmania* Kmp11 specific primers: 5′- GCCTGGATGAGGAGTTCAACA-3′ and 5′-GTGCTCCTTCATCTCGGG-3′on cDNA reverse-transcribed from total RNA extracted from footpad lysates. Briefly, footpads were homogenized using the Tissuelyzer (Qiagen), and total RNA was extracted using TRIzol reagent (Invitrogen). cDNA was synthesized using SuperScript II Reverse Transcriptase (Invitrogen), followed by purification using the QIAquick PCR purification Kit (Qiagen). Gene expression levels were analyzed using the LightCycler480 system (Roche Applied Science). Absolute parasite burden was determined using a standard curve for *Leishmania* Kmp11 gene content with a pre-determined number of *L. guyanensis* parasites.

### *In vitro* Restimulation of Lymph Node Cells and Cytokine Quantification

Draining popliteal lymph nodes were extracted from mice and stimulated in complete DMEM (Gibco) with a final concentration of 10% heat-inactivated FBS, 1% penicillin/streptomycin, 2 mM L-glutamine, 5×10^−5^ M of β-mercaptoethanol and 1% HEPES (Sigma-Aldrich) at 5×10^6^/ml cells with 1×10^6^/ml UV-irradiated *L. guyanensis* promastigotes. After 72 hours, levels of IFN-γ, IL-4, IL-10, and IL-13 (eBioscience) and IL12p70 (BD Biosciences) were quantified by ELISA in cell-free culture supernatants. In each case the manufacturer’s protocols were explicitly followed.

### Quantification of Cytokine Transcripts in the Draining Lymph Nodes of Infected Mice

RNA was extracted from draining popliteal lymph node cell suspensions using TRIZol reagent (Life technologies) and then clean and concentrated using appropriate columns from Zymoresearch. cDNA was reverse-transcribed and purified as described previously [3]. Relative quantification at the transcript level was performed by qRT-PCR using the Lightcycler480 system (Roche applied sciences) and results were calculated using the 2^−ΔΔCT^ method with Tata binding protein (Tbp) as a reference gene and normalized to give the average expression level of the WT C57BL/6 a value of 1. Gene specific primers used were the following: Tbp-5′-CCGTGAATCTTGGCTGTAAAC-3′, 3′-TCCAGAACTGAAAATCAACGC-5′; Il4-5′-CGGAGATGGATGTGCCAAAC-3′, 3′-AGCCCTACAGACGAGCTCACTC-5′; Il12p40-5′-GGAAGCACGGCAGCAGAATAA-3′, 3′-CTTGAGGGAGAAGTAGGAATG-5′; Il12p35-5′-AGGACTTGAAGATGTACCAG-3′, 3′-CATTCTGTGTGAGGAGGG-5′; Il13-5′-GAGGAGCTGAGCAACATC-3′, 3′-GTCAGGGAATCCAGGGCTA-5′; Il10-5′-ACCTGCTCCACTGCCTTGCT-3′, 3′-GGTTGCCAAGCCTTATCGGA-5′; IFN-γ-5′- GGATGCATTCATGAGTATTG-3′, 3′-GGAGTCCTTCGCCTTTTC-5′.

## Results

### MyD88 and TLR9 are Involved in the Development of Resistance to *L. guyanensis* Infection in Mice, Irrespective of LRV Presence

The ability of TLR9 and MyD88 to impact immune responses against *L.g.* LRV^high^ and *L.g.* LRV^low^ parasites was investigated using subcutaneous footpad infection in a murine model. These two isolates were derived from the M5313 strain of *L. guyanensis* based on reproducible disseminated lesion development in the Golden hamster model of infection [27]. The ability of these isolates to induce secondary lesion development could be reliant on their high LRV viral burden [3].

**Figure 1:**
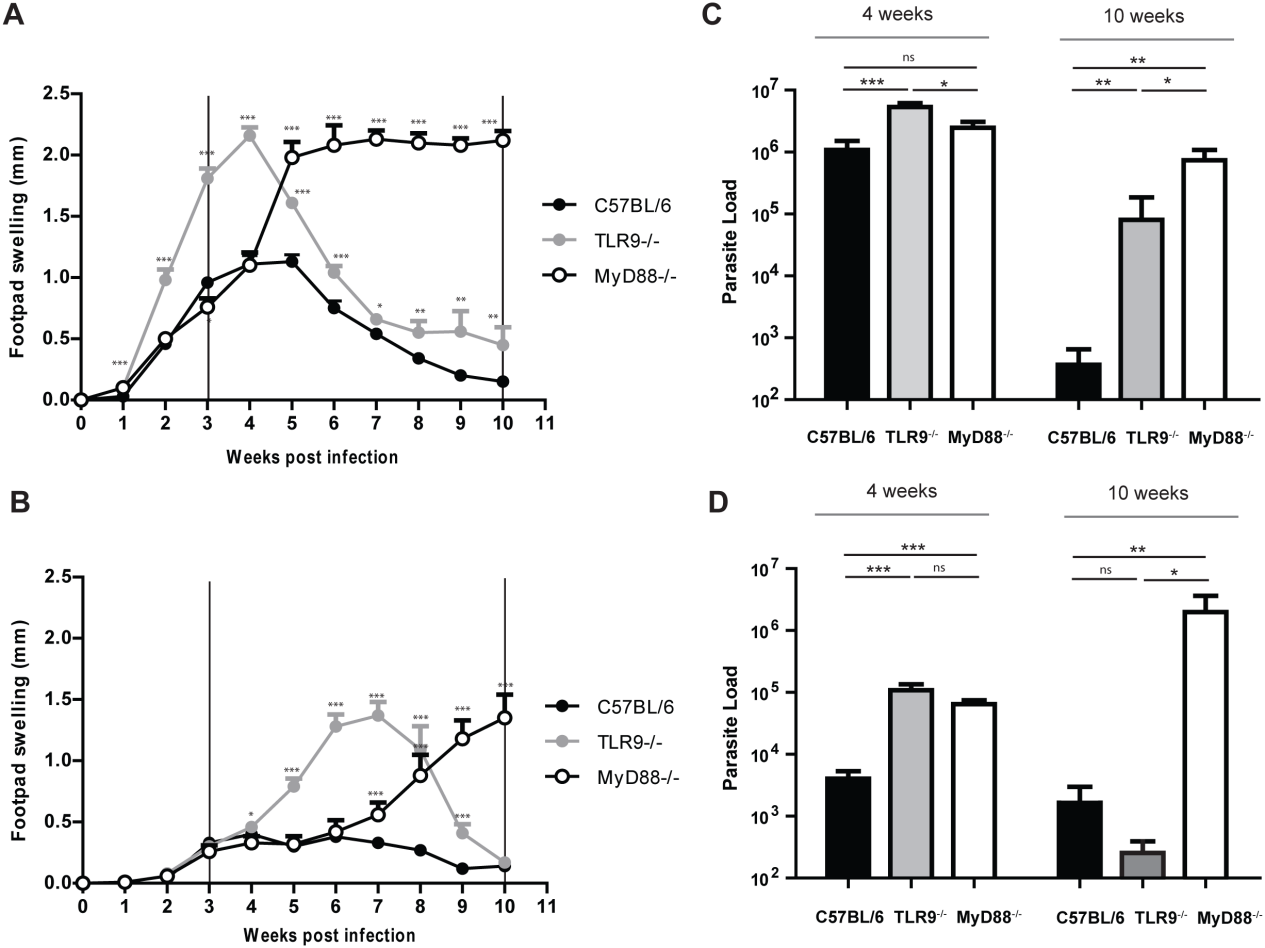
MyD88^−/−^ and TLR9^−/−^ mice are more susceptible to infection with *L. guyanensis*. Wild type C57BL/6, MyD88^−/−^ and TLR9^−/−^ mice (n≥4) were infected into the hind footpads. Footpad swelling of mice infected with either *L.g.* LRV^high^ (A) or *L.g.* LRV^low^ (B) was measured weekly over 10 weeks using a Vernier caliper. Parasite burden in footpads of *L.g.* LRV^high^ (C) or *L.g.* LRV^low^ (D) infected mice was determined at indicated time points by quantitative-real time PCR using parasite-specific *KMP11* primers. Results are expressed as the mean± SEM. Significance determined at *p≤0.05, **p≤0.01, ***p≤0.005.

Following infection, we could ascertain that *L. guyanensis* isolates, regardless of their LRV burden, displayed similar disease evolution phenotypes in MyD88^−/−^ and TLR9^−/−^ mice. More specifically, we observed that *L. guyanensis* infection in MyD88^−/−^ mice induced non-healing, progressive lesions that were associated with increased parasite load (Fig. 1). However, only the MyD88^−/−^ infected with the *L.g.* LRV^high^ developed sever ulcers at the footpad injection site (Fig. 1A). This increase in susceptibility was correlated to an increased parasite burden in the footpad (Fig. 1C–D). On the another hand, the TLR9^−/−^ mice displayed a transient increased peak in footpad swelling (Fig. 1A–B) that correlated with a transient increased in parasitemia at 4 weeks post infection (Fig. 1), and this after being infected either with *L.g.* LRV^low^ or *L.g.* LRV^high^ parasites. Interestingly, the *L.g.* LRV^high^ infected TLR9^−/−^ mice with resolved lesions retained a higher degree of parasitemia as compared to wild-type C57BL/6 mice at week 10 (Fig. 1C).

Additionally, we also analyzed the role of other MyD88-dependent TLRs and from our results we could exclude a likely role for TLR2, and TLR4 signaling pathway in resistance to *L. guyanensis* as these mice display similar footpad swelling profiles as wild-type mice following infection with either *L.g.* LRV^high^ or *L.g.* LRV^low^ parasites (Fig. S1).

### Increased Susceptibility of MyD88^−/−^ and TLR9^−/−^ Mice to *L. guyanensis* Infection is Associated with the Development of a Th2 Response

**Figure 2:**
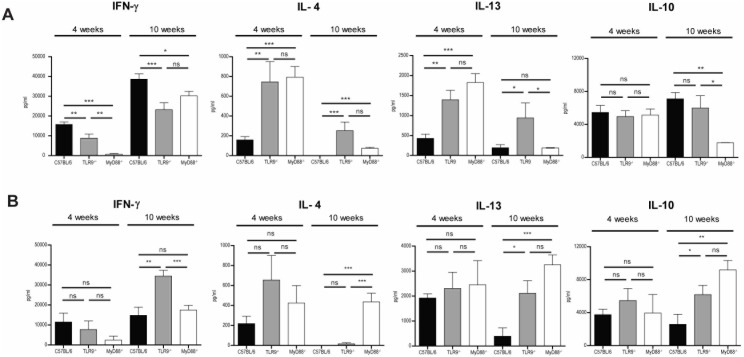
Absence of MyD88 and TLR9 signaling pathways results in a more Th2-polarized immune response. At specific time points, draining lymph node cells were restimulated with UV-irradiated *L. guyanensis* parasites with *L.g.* LRV^high^ (A) or *L.g.* LRV^low^ (B) for 72 hours and cytokine levels were quantified by ELISA. Results are expressed as mean ± SEM. Significance determined at *p≤0.05, **p≤0.01, ***p≤0.005.

Given the clear increase in susceptibility of both the MyD88^−/−^ and the TLR9^−/−^ mice infected with both strains of *L. guyanensis* parasites, the immune response within the draining lymph nodes (LN) of the mice was investigated. Analysis of cytokine production in supernatants of *L.g.* LRV^high^ stimulated LN cells revealed that MyD88^−/−^ or TLR9^−/−^ mice mounted a typical Th2 cell response with high levels of IL-4, IL-13 associated with a decrease in IFN-γ production comparison with control mice (at week 4 and 10) (Fig. 2A). *L.g.* LRV^low^infection in MyD88^−/−^ and TLR9^−/−^ mice, also induced Th2 immune responses as shown by high IL-13 and IL-4 production nevertheless this response was less pronounced than that of the same mice infected with *L.g.* LRV^high^ (Fig. 2B). Interestingly, TLR9^−/−^ mice infected with *L.g.* LRV^low^ produced significantly more IFN-γ at week 10 and this could be to counteract the earlier Th2 initiated response (Fig. 2B). Similar results were obtained by quantitative real time PCR (qRT-PCR) analysis on the draining lymph node (dLN) cells for Th1 and Th2 associated cytokines on all experimental mice groups (Fig. S2). This clearly demonstrates that MyD88 and TLR9 deficient mice have impaired immune response generation after infection with *L.guyanensis*, regardless of the presence of LRV virions.

### The Increased Susceptibility of Mice Infected with *L. guyanensis* Parasites is due to the Impaired Production IL-12

**Figure 3:**
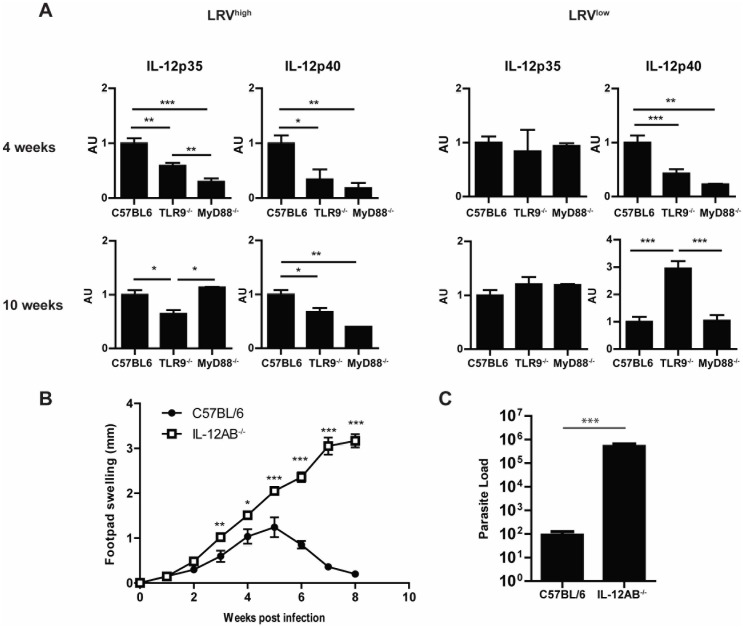
MyD88 and TLR9 are involved in IL-12 production following infection and their absence increases susceptibility to infection. At indicated time points RNA from draining popliteal lymph node cells of wild-type, TLR9^−/−^ and Myd88^−/−^ mice infected by *L.g.* LRV^high^ (Fig3A left panel) or *L.g.* LRV^low^ (Fig3A right panel) were reverse-transcribed into cDNA and relative transcript levels were determined using gene specific primers by quantitative real time PCR. Results are expressed as mean± SEM of the individual mice analyzed per group with the average value for the C57BL/6 mice given a value of 1. A.U refers to Arbitrary Units of fold change. (B) Mice (n≥4) were infected in the hind footpads and footpad swelling was measured weekly over 8 weeks using a Vernier caliper. (D) At 8 weeks post-infection mice were sacrificed and footpad parasite burden was determined using parasite specific Kmp11 primers. Results are expressed as the mean ± SEM. Significance determined at *p≤0.05, **p≤0.01, ***p≤0.005.

Given that previous reports have demonstrated that MyD88^−/−^ mice following infection with different *Leishmania* species have an impaired ability to produce IL-12, we tested whether this could also be the case in LRV harboring *L. guyanensis* infected mice. Firstly, we analyzed the transcript levels of IL-12p40 and IL-12p35 in the draining LN of *L.g.* LRV^high^ or *L.g.* LRV^low^ infected MyD88^−/−^ and TLR9^−/−^ mice as compared to controls. We observed that both the MyD88^−/−^ and the TLR9^−/−^ mice infected with *L.g.* LRV^high^ parasites had significantly diminished transcripts of IL-12p40 and IL-12p35 except for the latter in MyD88^−/−^ mice at 10 weeks post infection (Fig. 3A). In *L.g.* LRV^low^ infected mice, there was no consistent modulation of IL-12p35 as compared to WT mice at both time points analyzed (Fig. 3A), whilst IL-12p40 transcripts were diminished in MyD88^−/−^ and TLR9^−/−^mice only at 4 weeks post infection (Fig. 3A). As modulation at the RNA level does not necessarily reflect those at the protein level, we attempted to quantify the amount of IL-12p40 protein in the culture supernatant by ELISA but the levels were below the detection limit (data not shown).

Finally, the essential role of IL-12 in the development of resistance to infection was tested by analyzing the disease evolution profile and development of a Th1 immune response in IL-12AB^−/−^ mice (that do not express functional IL-12) in infections with *L.g.* LRV^high^ parasites. As with infections with other *Leishmania strains*, IL12AB^−/−^ mice were completely susceptible to infection, resulting in progressive, ulcerating lesion development and increased parasite burden (Fig. 3B and 3C). This necessitated the sacrifice of these mice at week 8 and confirmed that IL-12 was sufficient for the development of resistance to infection in C57BL/6 mice infected with *L. guyanensis* parasites, even when carrying a high burden of LRV.

## Discussion

The purpose of this study was to elucidate the role of MyD88 and TLR9 in disease progression and immune response generation in mice infected with *L. guyanensis*, and to determine if a high *Leishmania RNA virus* (LRV) burden within promastigotes could impair these protective immune responses.

Our experimental data clearly supported a role for the innate immune system and MyD88-dependent signaling pathways in the development of resistance to infection with *L. guyanensis*, and also implied a partial role for the MyD88 dependent, TLR9 signaling pathway. The results show that protective immune responses elicited via MyD88 and TLR9 arise independently to those mediated by TLR3 and viral dsRNA recognition. As evidenced by the fact that infection with either *L.g* LRV^high^ or *L.g* LRV^low^ parasites into the MyD88^−/−^ or TLR9^−/−^ mice were more susceptible than wild type controls regardless of the viral burden. However, presence of a high LRV burden does influence overall disease severity, where all mice infected with *L.g.* LRV^high^ showed an increased footpad swelling peak as compared to *L.g.* LRV^low^. This increase in susceptibility is reliant on TLR3 recognition of viral dsRNA that seems to modulate Th1/Th2 immune response polarization or to impaired parasite killing mechanisms.

As with other studies, we observed that the susceptible disease phenotype was more severe in MyD88^−/−^ mice than in TLR9^−/−^, and implies that other TLRs (that utilize MyD88) could participate in the disease resolution and elimination of parasites. Currently, we can exclude a role for TLR7 and the cell membrane localized TLR4 and TLR2, as mice deficient for these receptors do not display any discernable difference in disease phenotype in either the *L.g* LRV^high^ or *L.g* LRV^low^ infected mice [3].

In this study, we specifically demonstrated that the loss of MyD88 and TLR9 signaling pathways resulted in a modulation of the Th1/Th2 balance, with a significant increase in Th2 associated cytokines (IL-4 and IL-13) in the draining lymph nodes of both MyD88^−/−^ and TLR9^−/−^ mice. This Th2-polarized immune response could also be correlated with a diminished production of IL-12p40 and IL-12p35 transcripts *ex vivo,* following infection, which we also confirmed was essential for healing and elimination of parasites. Although other studies have demonstrated a role for both MyD88 and TLR9 in the generation of IL-12 and IFN-γ-dependent protective immune responses murine models of infection with other *Leishmania* parasites (specifically *L. major, L. donovani* and *L.(V) braziliensis* [13]–[15], [17], [19]–[21]) this study demonstrates that the viral immune response driven by LRV does not impair protection conferred by MyD88 and TLR9 pathways nor those elicited due to IL-12.

Although there is currently no vaccine available against leishmaniasis, the past has shown us that protection is, indeed, possible. Leishmanization - the injection of live parasites into an individual to induce protection- was a common practice in the Middle East but adverse events such as uncontrolled skin lesions, HIV-co-infections, and variability in protection limited it potential application on a large scale. Thus, several different vaccine strategies have been investigated in the pursuit of the anti-*Leishmania* vaccine, including whole organism formulations using live-attenuated or killed organisms, and defined vaccines targeting *Leishmania* specific motifs such as the LACK antigen or DNA-based vaccines. Unfortunately, however, poor immunogenicity and other factors have limited their success.

Given this limitation, CpG based motifs (that are recognized by TLR9) and other MyD88 stimulating ligands have been investigated for their potential as adjuvants to boost the development of protective immune responses against *Leishmania* parasites [28], [29]. Studies have shown that regardless of the vaccine antigen used CpG-oligodeoxynucleotides (CpG-ODN) enhanced the protection of mice subsequently challenged with the parasites. Specifically, vaccination of susceptible BALB/c mice with soluble *Leishmania* antigen (SLA) and CpG provided enhanced protection in challenged mice that persisted for over 6 months [30]. This protection was linked with the development of memory CD4 and CD8 T cell immune responses [31]. Given that TLR9 responses remain protective regardless of the LRV burden in infecting parasites, CpG-based adjuvants stand as a promising addition for vaccines serving regions that are endemic to parasites of mixed LRV-content.

It is important to note, however, that TLR-stimulation can result in potent inflammatory responses that could exacerbate severity of *Leishmania* infections, as we previously reported for the pathogenic TLR3 stimulation by high viral dsRNA burden in *L. guyanensis* [3]. Thus, further experimentation is required to gauge the effects of all TLR-based adjuvants on infections with parasite species to better tailor their therapeutic use in leishmaniasis.

## Supporting Information

Figure S1 **TLR2** ^−/−^ **and TLR4** ^−/−^ **mice display no difference in footpad swelling following** ***L. guyanensis*** **infection.** Mice (n≥5) were infected into the hind footpads with either *L.g.* LRV^high^ or *L.g.* LRV^low^, and footpad swelling was measured weekly over 10 weeks using a vernier caliper.
(TIF)

Figure S2 **Absence of MyD88 and TLR9 signaling pathways increases transcripts of Th2 cytokines.** At specific time points RNA from draining popliteal lymph node cells of wild-type, TLR9^−/−^ and MyD88^−/−^ mice infected by *L.g* LRV^high^ (A) or *L.g* LRV^low^ (B) were reverse-transcribed into cDNA and relative transcript levels were determined using gene specific primers by quantitative real time PCR. Results were expressed as mean± SEM of the individual mice analyzed per group with the average value for the C57BL/6 mice given a value of 1. Tbp was used as a reference gene. A.U.: arbitrary units corresponding to fold changes in expression. Significance determined at *p≤0.05, **p≤0.01, ***p≤0.005.
(TIF)
