# Supplementary figures and images for: MyD88 and TLR9 Dependent Immune Responses Mediate Resistance to Leishmania guyanensis Infections, Irrespective of Leishmania RNA Virus Burden
Source: PLoS One. 2014 May 6;9(5):e96766. doi: 10.1371/journal.pone.0096766 (PMC4011865; doi:10.1371/journal.pone.0096766)

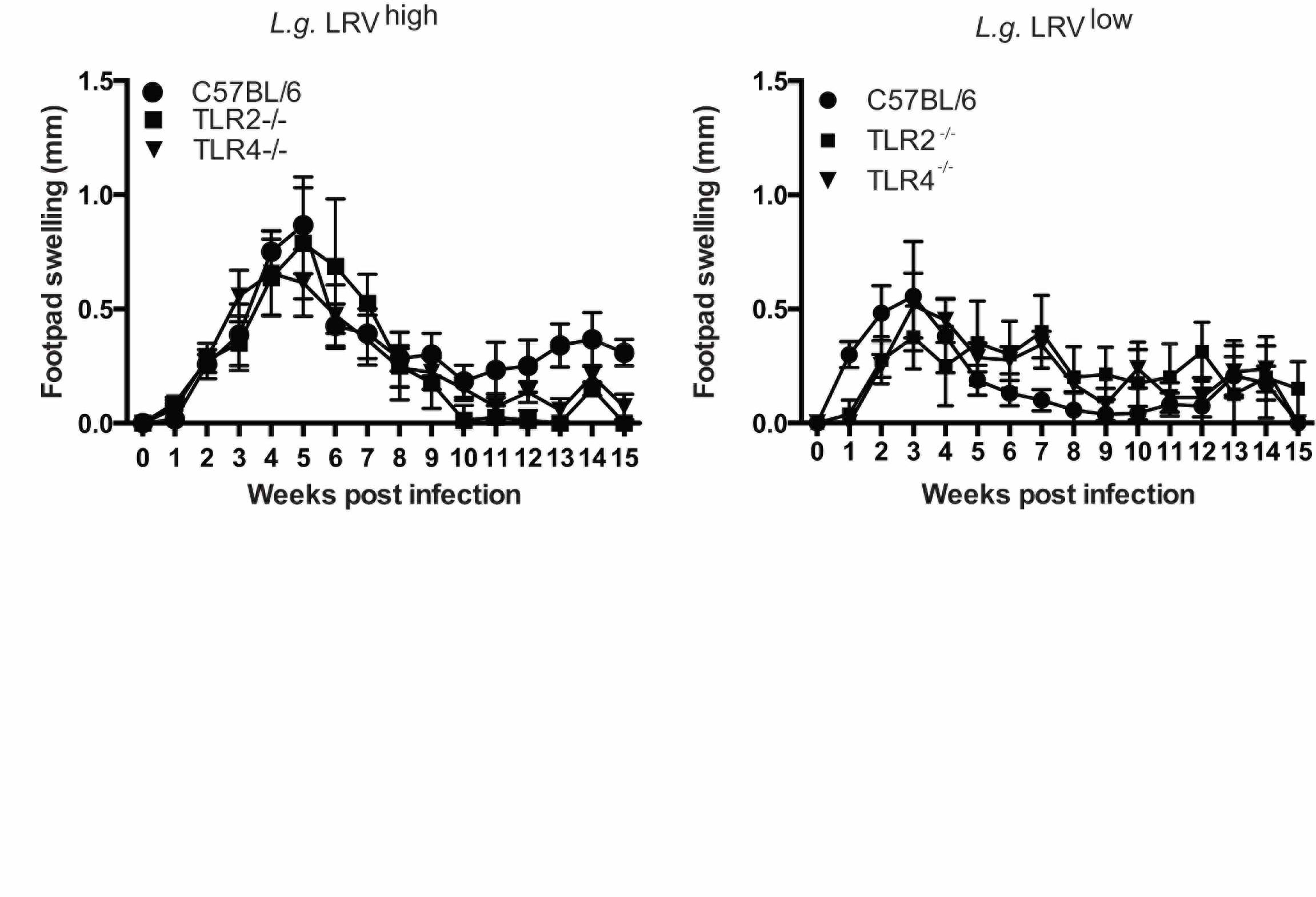

Supplement: Figure S1 — TLR2 −/− and TLR4 −/− mice display no difference in footpad swelling following L. guyanensis infection. Mice (n≥5) were infected into the hind footpads with either L.g. LRVhigh or L.g. LRVlow, and footpad swelling was measured weekly over 10 weeks using a vernier caliper. (TIF) [file pone.0096766.s001.tif]

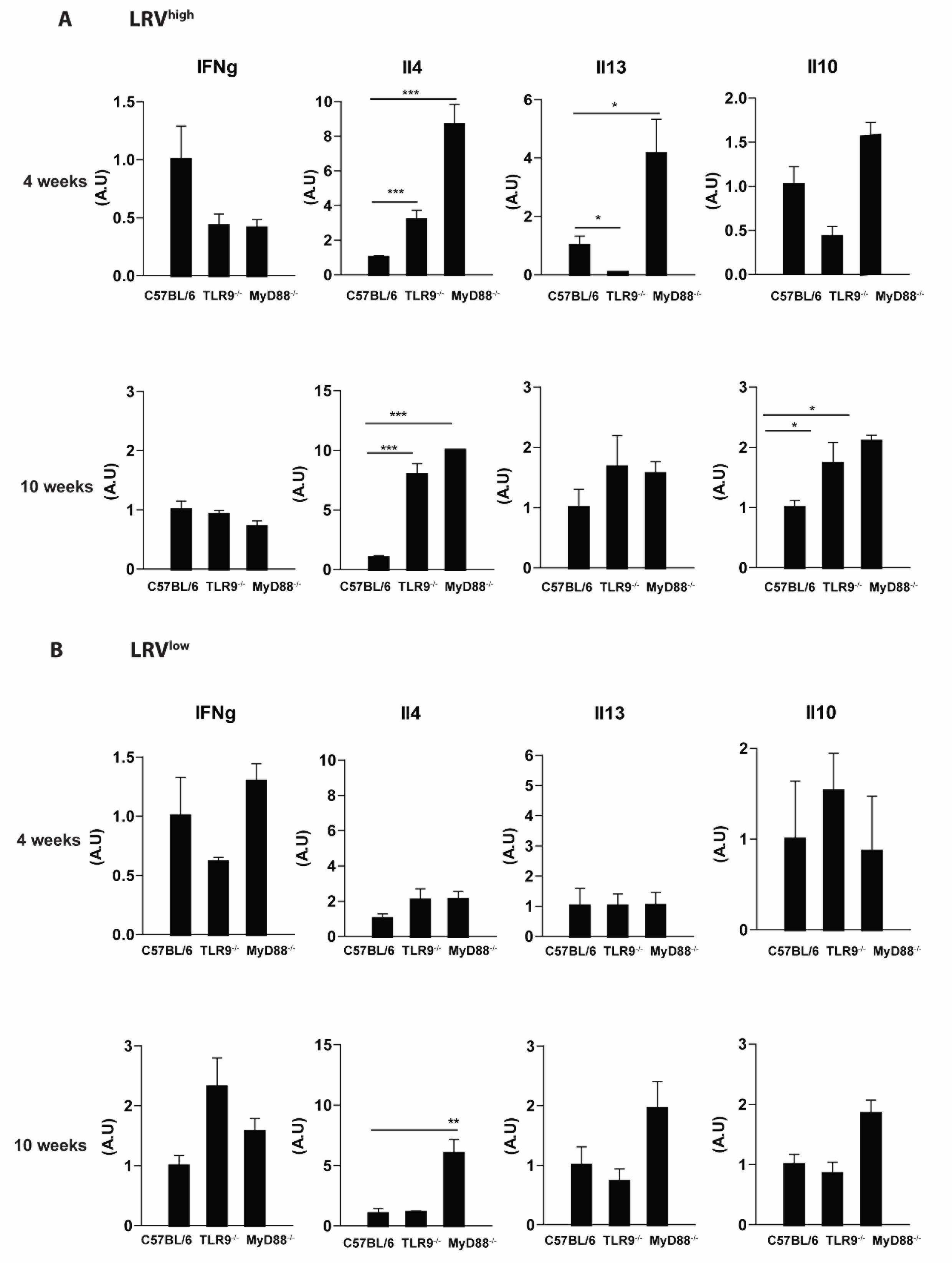

Supplement: Figure S2 — Absence of MyD88 and TLR9 signaling pathways increases transcripts of Th2 cytokines. At specific time points RNA from draining popliteal lymph node cells of wild-type, TLR9−/− and MyD88−/− mice infected by L.g LRVhigh (A) or L.g LRVlow (B) were reverse-transcribed into cDNA and relative transcript levels were determined using gene specific primers by quantitative real time PCR. Results were expressed as mean± SEM of the individual mice analyzed per group with the average value for the C57BL/6 mice given a value of 1. Tbp was used as a reference gene. A.U.: arbitrary units corresponding to fold changes in expression. Significance determined at *p≤0.05, **p≤0.01, ***p≤0.005. (TIF) [file pone.0096766.s002.tif]
